# A simple daily dynamic feeding regimen for reducing phosphorus consumption and excretion in laying hens

**DOI:** 10.1016/j.aninu.2022.07.003

**Published:** 2022-07-22

**Authors:** Xujie Liao, Jiakun Yan, Jionghao Chen, Zhenyu Huang, Tianshuai Xiao, Changqing Li, Chong Pan, Xin Yang, Yanli Liu, Thomas D. Crenshaw, Xiaojun Yang, Zhouzheng Ren

**Affiliations:** a College of Animal Science and Technology, Northwest A&F University, Yangling, Shaanxi 712100, China; b Department of Animal Sciences, University of Wisconsin-Madison, Madison, WI 53706, USA

**Keywords:** Phosphorus-calcium homeostasis, Dynamic feeding regimen, Egg production, Laying hen, Phosphorus excretion

## Abstract

Phosphorus metabolism in laying hens is a highly dynamic process over the course of the 24 h egg-laying cycle. Adjusting the phosphorus feeding regimen according to the daily egg-laying cycle may help to improve phosphorus utilization efficiency. Hy-Line Brown layers (*n* = 120; 70 wk old) were offered 4 different phosphorus daily regimens: (1) RR, fed regular phosphorus at both 09:00 and 17:00; (2) RL, fed regular phosphorus at 09:00 and low phosphorus at 17:00; (3) LR, fed low phosphorus at 09:00 and regular phosphorus at 17:00; (4) LL, fed low phosphorus at both 09:00 and 17:00. The regular and low phosphorus diets contained 0.32% and 0.14% non-phytate phosphorus, respectively. The feeding trial lasted for 12 wk. As a result, layers on the RL regimen had decreased laying rate (*P* < 0.05; 5 to 8, 9 to 12, and 1 to 12 wk) when compared to all other regimens. Layers on the LL regimen had decreased eggshell thickness and specific gravity (*P* < 0.05; wk 8) when compared to all other regimens, and had decreased egg shell strength (*P* < 0.05; wk 8) when compared to RL and LR regimens. When compared to the RR regimen (a common practice in the industry), layers on the LR regimen had: (1) identical laying performance and egg quality (*P* > 0.05); (2) decreased phosphorus excretion (*P* < 0.05) during the period of 09:00 to 17:00; (3) increased jejunal calbindin D28k protein expression (*P* < 0.05) 2 h after feeding in the morning; (4) decreased serum fibroblast growth factor 23 and calcitriol levels (*P* < 0.05), decreased jejunal type III sodium-phosphate cotransporter 2 gene and protein expression (*P* < 0.05), and decreased renal type III sodium-phosphate cotransporter 1 protein expression (*P* < 0.05), 2 h after feeding in the afternoon. In summary, when dietary phosphorus was supplemented in accordance with daily serum phosphorus rhythms (i.e., the LR regimen), laying performance and egg quality were well supported whilst significantly decreasing phosphorus consumption and excretion. Thus, serum phosphorus rhythms will need to be carefully maintained when developing dietary phosphorus-reduction strategies in laying hens.

## 1 Introduction

Phosphorus in excreta is a known pollutant which may cause algal blooms in surface waters near intensive poultry farms (Mekonnen and Hoekstra, 2018). Discovery of a novel means to reduce the consumption and excretion of phosphorus is therefore of interest to improve the environment and enhance the sustainability of the egg production industry. Current solutions for mitigating the phosphorus footprint of laying hens mainly involve technologies (e.g., phytase) that target intestinal phosphorus digestion and absorption processes (Li et al., 2015; Ren et al., 2017). However, simply improving phosphorus influx efficiency does not necessarily mean phosphorus will be retained, because the laying hen needs to rapidly adjust the influx-efflux balance of body phosphorus based on the daily cycle of egg formation (Martindale, 1969).

Phosphorus metabolism in laying hens is highly dynamic over the course of the 24 h egg-laying cycle (Ren et al., 2019). During the eggshell formation period, which mainly happens in the evening and night, calcium and phosphorus stores are released from medullary bone (Whitehead, 2004). The released phosphorus is mainly accumulated in the circulation as eggshell calcification sequesters mostly calcium, but very little phosphorus (Prashad and Edwards, 1973). As a result, the increase in blood phosphorus induces secretion of fibroblast growth factor 23 (FGF23), a bone-sourced hormone that strongly stimulates body phosphorus loss (Hadley et al., 2016; Hernando et al., 2021). Thus, the laying hen is in a state of phosphorus-efflux during the period of eggshell calcification. After the eggshell formation is completed in the early morning, medullary bone resorption ceases and reconstruction occurs (Van de Velde et al., 1985). Accordingly, serum phosphorus and FGF23 concentrations are reduced, and the laying hen switches to a phosphorus-influx mode during the subsequent daylight period (Hadley et al., 2016). By understanding the dynamics of phosphorus metabolism in the laying hen, we hypothesized that adjusting the phosphorus feeding regimen according to the daily egg-laying cycle would improve the phosphorus utilization efficiency.

In conventional feeding systems, laying hens are provided with a single diet with a constant phosphorus level throughout the entire day (MolnÁR et al., 2018). In this study, 2 diets with different phosphorus levels were alternately presented to the laying hens to meet the specific phosphorus requirement during different stages of the daily egg-laying cycle. A number of analyses were completed to evaluate the effects of dietary phosphorus feeding regimens on egg production performance, phosphorus excretion, and physiological and molecular biological parameters for the body phosphorus homeostasis. Our objectives were to provide a simple daily dynamic feeding regimen for reducing phosphorus consumption and excretion in laying hens and to examine the underlying mechanisms.

## 2 Materials and methods

### 2.1 Animal ethics

All animal procedures described were approved (protocol number DK2021032) by the Animal Ethics and Welfare Committee of the Northwest A&F University (Yangling, Shaanxi, China).

### 2.2 Experimental design and sample collection

Hy-Line Brown laying hens were individually housed in cages (depth, 45 cm; width, 35 cm; height, 45 cm) with raised wire floors at the Animal Nutrition & Healthy Feeding Research Laboratory (Northwest A&F University, Yangling, Shaanxi, China). A photoperiod of 16 h of light:8 h of dark was applied (lights-on, 05:30; lights-off, 21:30). The layers were fed twice daily, at 09:00 and 17:00, ad libitum intake (allowing for approximately 5% feed refusal). For each feeding time point, the feeder was cleaned and refilled manually. At 70 wk of age, a total of 120 layers were randomly selected and assigned to 4 phosphorus feeding regimens each with 6 replicates of 5 hens: (1) RR, fed with a regular phosphorus diet at both 09:00 and 17:00; (2) RL, fed with a regular phosphorus diet at 09:00 and a low phosphorus diet at 17:00; (3) LR, fed with a low phosphorus diet at 09:00 and a regular phosphorus diet at 17:00; (4) LL, fed with a low phosphorus diet at both 09:00 and 17:00. The average body weight of the 70 wk laying hens was 2,113 ± 184 g, with no statistical difference among treatments (RR, 2,120 ± 159 g; RL, 2,151 ± 211 g; LR, 2,088 ± 206 g; LL 2,093 ± 159 g; *P* > 0.05). The laying hens were housed at the Animal Nutrition & Healthy Feeding Research Laboratory and fed with the regular phosphorus diet since 60 wk of age. The study followed a randomized complete block design. The 120 experimental cages were divided into 6 blocks based on location within the battery. Each block contained 4 pens, and each pen contained 5 cages. The four diets were randomized within each block so that each diet was fed to the 5 birds of each pen. The regular phosphorus diet contained 0.32% non-phytate phosphorus, the recommended phosphorus requirement for laying hens according to the Chinese Feeding Standard of Chicken (NY/T 33-2004). The low phosphorus diet contained 0.14% non-phytate phosphorus, the basal phosphorus composition of the corn-soybean meal-based laying-hen diet with no inorganic phosphorus supplementation. The levels of calcium carbonate and zeolite powder were adjusted accordingly to make sure both regular and low phosphorus diets had the same level of calcium (3.50%, recommended by the Chinese Feeding Standard of Chicken NY/T 33-2004). Both diets did not contain an exogenous phytase. Ingredient and nutrient composition of the experimental diets are presented in Table 1. The feeding trial lasted for 12 wk.

**Table 1:** Ingredients and nutrient composition of experimental diets (%, as-fed basis).

Item	Regular phosphorus	Low phosphorus
Ingredients		
Corn	56.69	56.69
Soybean meal, 43%	25.77	25.77
Distillers dried grains with solubles	4.00	4.00
Calcium carbonate	9.04	9.73
Dicalcium phosphate	1.15	0.00
Soybean oil	1.51	1.51
Sodium chloride	0.26	0.26
DL-Methionine, 98.5%	0.18	0.18
Choline chloride, 60%	0.15	0.15
Zeolite powder	0.25	0.71
Premix^1^	1.00	1.00
Total	100.00	100.00
Nutrient levels		
Metabolizable energy, kcal/kg (formulated)	2,600	2,600
Crude protein (formulated/analyzed)	16.50/16.29	16.50/16.26
Calcium (formulated/analyzed)	3.50/3.47	3.50/3.52
Total phosphorus (formulated/analyzed)	0.53/0.49	0.35/0.34
Phytate phosphorus (formulated/analyzed)	0.21/0.17	0.21/0.19
Non-phytate phosphorus (formulated/calculated^2^)	0.32/0.32	0.14/0.15

1 Provided per kilogram of diet: 80 mg zinc (from ZnSO_4_·H_2_O); 60 mg manganese (from MnSO_4_·H_2_O); 8 mg copper (from CuSO_4_·5H_2_O); 0.35 mg iodine (from Ca(IO_3_)_2_); 0.3 mg selenium (from Na_2_SeO_3_); 8,000 IU vitamin A (from retinyl palmitate); 1,600 IU vitamin D_3_ (from cholecalciferol); 30 mg vitamin E (from tocopheryl acetate); 1.5 mg vitamin K_3_ (from menadione); 4 mg vitamin B_1_ (from thiamine hydrochloride); 13 mg vitamin B_2_ (from riboflavin); 20 mg vitamin B_3_ (from nicotinamide); 15 mg vitamin B_5_ (from calcium D-pantothenate); 6 mg vitamin B_6_ (from pyridoxine hydrochloride); 0.15 mg vitamin B_7_ (from biotin); 1.5 mg vitamin B_9_ (from folic acid); 0.02 mg vitamin B_12_ (from cobalamin).

2 Calculated using analyzed levels of total phosphorus and phytate phosphorus.

Laying performance (measured as laying rate, egg weight, daily feed intake and feed-to-egg ratio) was recorded/calculated weekly. On the day before the start of the feeding trial, and on the last day of wk 4, 8 and 12, 2 eggs per replicate were randomly selected for egg quality analysis. Briefly, shell thickness was analyzed using an ultrasonic gauge (ETG-1061; Robotmation, Co., Ltd., Tokyo, Japan); shell strength was analyzed using a texture analyzer (EFG-0503; Robotmation, Co., Ltd., Tokyo, Japan); yolk pigmentation and Haugh units were analyzed using an Egg Multi-Tester (EMT-5200; Robotmation, Co., Ltd., Tokyo, Japan); specific gravity was analyzed using the flotation method with sodium chloride solutions varying in specific gravity from 1.070 to 1.110 g/cm^3^; and shell index was calculated as shell weight/egg weight × 100%.

On the penultimate day of the feeding trial, two layers per replicate were randomly selected for excreta collection. The total excreta samples were collected for 24 h, divided into 2 periods: (1) total collections from 09:00 to 17:00; and (2) total collections from 17:00 to 09:00 (next morning). On the last day of the feeding trial: (1) at 8:30, the hens that laid eggs were tagged; (2) of them, 2 hens per replicate were randomly selected for blood and tissue samples (one was sampled at 11:00, 2 h after feeding in the morning; the other one was sampled at 19:00, 2 h after feeding in the afternoon) (3) the sampling took about 1 h (i.e. from 11:00 to 12:00 in the morning, and from 19:00 to 20:00 in the afternoon); and the hens were sampled by order of block. The layers were bled (3 mL, collected from the wing veins) for serum and euthanized (via cervical dislocation) for tissues including jejunal mucosa, kidney and tibiotarsus.

### 2.3 Serum biochemical analysis

The concentrations of phosphorus and calcium and the activities of alkaline phosphatase (AKP) in serum samples were determined as previously described (Ren et al., 2018). Briefly, serum phosphorus analysis was based on the formation of 12-molybdophosphoric acid (phosphorus-molybdate reaction) and its reduction to a blue heteropoly compound. Serum calcium analysis was based on the formation of a deep-blue calcium-methyl thymol complex. Serum AKP analysis was based on the production of phenol (AKP decomposes disodium phenyl phosphate) which reacts with 4-aminoantipyrine to produce a red complex of quinone derivative. One unit of AKP activity (a King unit) was defined as the amount of AKP that catalyzes the formation of 1 mg phenol at 37 °C during a reaction period of 15 min. The concentrations of fibroblast growth factor-23 (FGF23), calcitriol and parathyroid hormone (PTH) in serum samples were analyzed using sandwich ELISA assays (Ren et al., 2020b). The above-mentioned colorimetric reactions were quantified using either UV-1800 spectrophotometer (Shimadzu, Japan; for serum phosphorus) or Synergy HT microplate reader (BioTek, Winooski, VT; for serum calcium, AKP, FGF23, calcitriol and PTH).

### 2.4 Excreta and tibiotarsus analysis

The samples were pre-treated (excreta samples, oven-dried; tibiotarsus samples, cleaned of muscle and articular cartilage, oven-dried and defatted), ashed, and acid digested for the determination of phosphorus (molybdate method) and calcium (ethylenediamine tetraacetic acid method) concentrations as previously described (Nie et al., 2013). The excreta samples collected from each 12 h (from 09:00 to 17:00; and from 17:00 to 09:00 next morning) were analyzed separately. The results were expressed on oven-dried basis for excreta samples and expressed on ash basis for tibiotarsus samples.

### 2.5 Western blotting

Jejunal mucosa and kidney samples were subjected to Western blotting procedures previously described in Ren et al. (2020b). Protein expressions of type IIa sodium-phosphate cotransporter (NPT2a), type IIb sodium-phosphate cotransporter (NPT2b), type III sodium-phosphate cotransporter 1 (PIT1), type III sodium-phosphate cotransporter 2 (PIT2), and calbindin D28k (CALB1) were analysed and normalized to beta actin (ACTB). Sources and concentrations of the primary and secondary antibodies are provided in Appendix Table 1. The protein bands were visualized by an imaging system (DNR, Micro Chemi, Israel) and quantified using the Image J software (CA, USA).

### 2.6 qPCR analysis

The mRNA expressions of *NPT2a*, *NPT2b*, *PIT1*, *PIT2*, and *CALB1* were analyzed in jejunal mucosa and/or kidney samples. The qPCR procedures were previously described in Ren et al. (2020a). Primer sequences for the target genes were designed using the Primer3 online program and are presented in Appendix Table 2. Relative expressions for target genes in a given tissue were calculated (2^−ΔΔCt^ method) using *ACTB* as a reference gene.

### 2.7 Data analysis

One-way ANOVA (SPSS software version 23.0, IBM Corp., Chicago, IL) was used to detect the differences among the four different phosphorus feeding regimens under a randomized complete block design. For laying performance, the statistical unit was each pen (containing 5 cages, *n* = 6). For egg quality parameters, the statistical unit was each egg (*n* = 12). For phosphorus and calcium excretion, the statistical unit was each selected hen (*n* = 12). For the other measurements, the statistical unit was each sampled hen (*n* = 6). Duncan's multiple range test was applied when significance was detected in one-way ANOVA. All statistical tests were considered significant at *P* ≤ 0.05. The results are presented as means and standard errors of the mean.

## 3 Results

### 3.1 Laying performance and egg quality

**Fig. 1:**
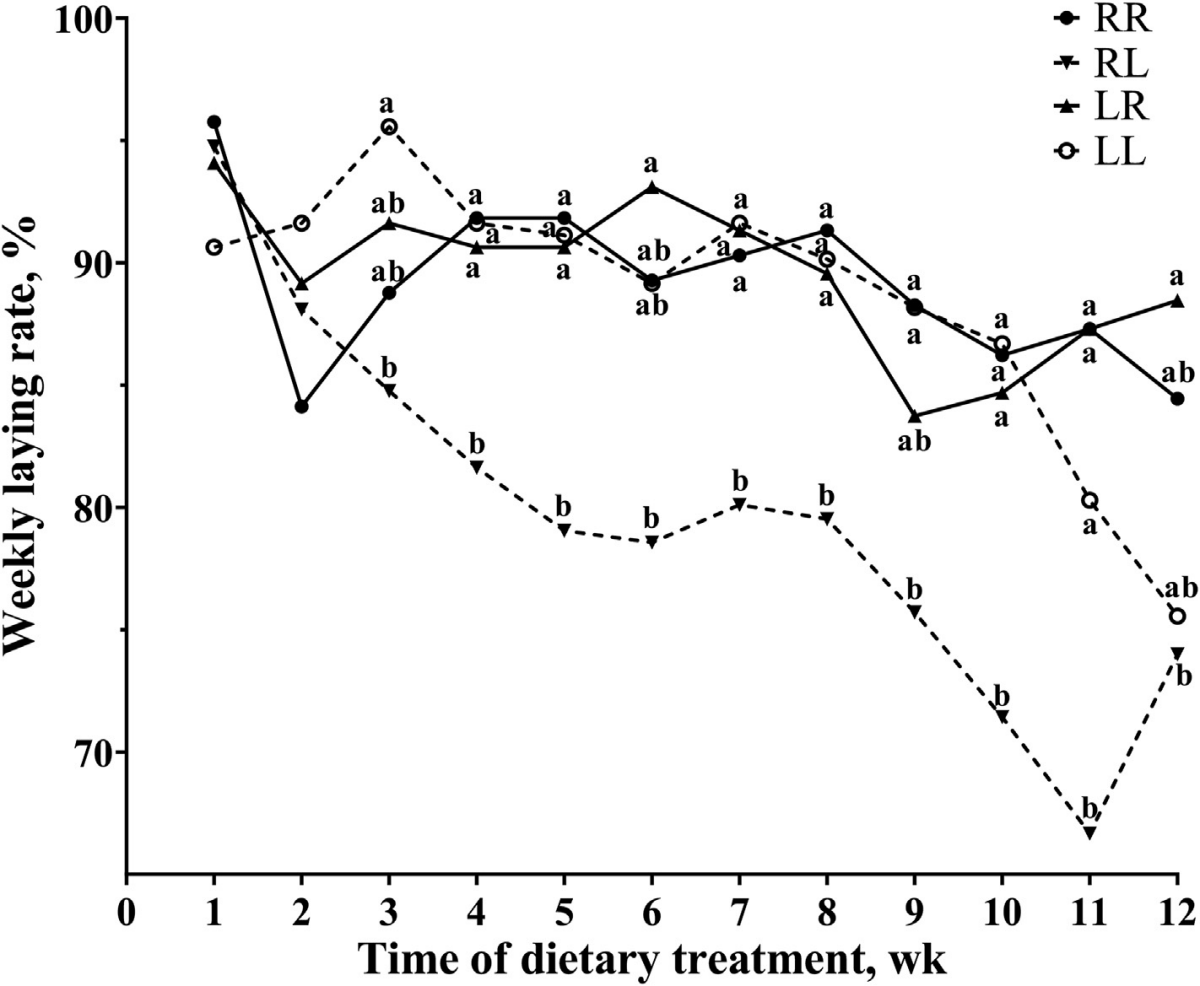
Effect of phosphorus feeding regimens on weekly laying rate of Hy-Line Brown layers. Four phosphorus feeding regimens: (1) RR, regular phosphorus at both 09:00 and 17:00; (2) RL, regular phosphorus at 09:00 and low phosphorus at 17:00; (3) LR, low phosphorus at 09:00 and regular phosphorus at 17:00; (4) LL, low phosphorus at both 09:00 and 17:00. ^a, b^ Values with no common superscripts differ significantly (*P* < 0.05).

Layers on the RL phosphorus feeding regimen had decreased laying rate (*P* < 0.05) at intervals from 5 to 8 wk, from 9 to 12 wk and from 1 to 12 wk when compared to those on the regimens of RR, LR and LL (Table 2). Weekly analysis showed that the reduction of laying rate in the RL regimen layers was first recorded in the third week and remained consistently lower throughout the feeding trial (Fig. 1). Phosphorus feeding regimens had no effects (*P* > 0.05) on egg weight, daily feed intake and feed-to-egg ratio.

**Table 2:** Effect of phosphorus feeding regimens on laying performance of Hy-Line Brown layers.

Item	Time of dietary treatment, wk	Phosphorus feeding regimen^1^				SEM	*P*-value
		RR	RL	LR	LL		
Laying rate, %	1–4	90.1	87.4	91.4	92.4	0.7	0.068
	5–8	90.7^a^	79.3^b^	91.2^a^	90.5^a^	0.9	<0.001
	9–12	86.6^a^	72.0^b^	86.0^a^	82.8^a^	1.2	<0.001
	1–12	89.1^a^	79.5^b^	89.6^a^	88.6^a^	0.6	<0.001
Egg weight, g	1–4	63.7	64.1	63.1	64.3	0.3	0.607
	5–8	65.4	65.1	64.9	65.8	0.4	0.906
	9–12	66.1	66.6	67.0	67.8	0.4	0.569
	1–12	65.1	65.3	65.0	66.0	0.3	0.588
Daily feed intake, g	1–4	112.0	112.4	114.4	113.5	1.0	0.830
	5–8	130.2	129.5	134.3	131.0	1.4	0.641
	9–12	134.6	137.6	137.4	132.4	1.3	0.478
	1–12	125.6	126.5	128.7	125.6	1.3	0.840
Feed-to-egg ratio, g:g	1–4	1.76	1.75	1.81	1.77	0.01	0.368
	5–8	2.00	1.99	2.07	1.99	0.02	0.479
	9–12	2.04	2.07	2.05	1.95	0.02	0.226
	1–12	1.93	1.94	1.98	1.90	0.02	0.502

^a, b^ Values in a row with no common superscripts differ significantly (*P* < 0.05).

1 Four phosphorus feeding regimens: (1) RR, regular phosphorus at both 09:00 and 17:00; (2) RL, regular phosphorus at 09:00 and low phosphorus at 17:00; (3) LR, low phosphorus at 09:00 and regular phosphorus at 17:00; (4) LL, low phosphorus at both 09:00 and 17:00.

As shown in Table 3, layers on the LL phosphorus feeding regimen had the following: (1) decreased egg shell thickness (*P* < 0.05) at wk 8 when compared to those on the regimens of RR, RL and LR; (2) decreased egg shell strength (*P* < 0.05) at wk 8 when compared to those on the regimens of RL and LR; and (3) decreased egg specific gravity (*P* < 0.05) at wk 8 when compared to those on the regimens of RR, RL and LR, and at wk 12 when compared to those on the regimens of RR and LR. Phosphorus feeding regimens had no effects (Appendix Table 3, *P* > 0.05) on shell index, egg yolk pigmentation and Haugh units. There was no difference in baseline egg quality measurements (eggs collected on the day before the start of the feeding trial) among treatments (Table 3 and Appendix Table 3).

**Table 3:** Effect of phosphorus feeding regimens on egg quality of Hy-Line Brown layers.

Item	Time of dietary treatment, wk	Daily phosphorus regimen^1^				SEM	*P*-value
		RR	RL	LR	LL		
Shell thickness, mm	0	0.388	0.402	0.407	0.390	0.003	0.129
	4	0.382	0.410	0.407	0.380	0.005	0.074
	8	0.389^a^	0.413^a^	0.399^a^	0.361^b^	0.005	0.001
	12	0.408	0.396	0.394	0.384	0.005	0.322
Shell strength, kg/cm^2^	0	48.9	45.8	45.8	45.6	0.9	0.529
	4	42.0	45.1	48.5	47.0	1.4	0.397
	8	39.3^ab^	46.4^a^	47.0^a^	32.8^b^	1.6	0.002
	12	44.9	46.5	47.0	36.7	1.6	0.063
Specific gravity	0	1.089	1.090	1.090	1.088	0.001	0.175
	4	1.092	1.094	1.097	1.094	0.001	0.184
	8	1.098^a^	1.099^a^	1.098^a^	1.093^b^	0.001	0.003
	12	1.095^a^	1.092^ab^	1.096^a^	1.089^b^	0.001	0.042

^a, b^ Values in a row with no common superscripts differ significantly (*P* < 0.05).

1 Four phosphorus feeding regimens: (1) RR, regular phosphorus at both 09:00 and 17:00; (2) RL, regular phosphorus at 09:00 and low phosphorus at 17:00; (3) LR, low phosphorus at 09:00 and regular phosphorus at 17:00; (4) LL, low phosphorus at both 09:00 and 17:00.

### 3.2 Phosphorus and calcium excretion

**Fig. 2:**
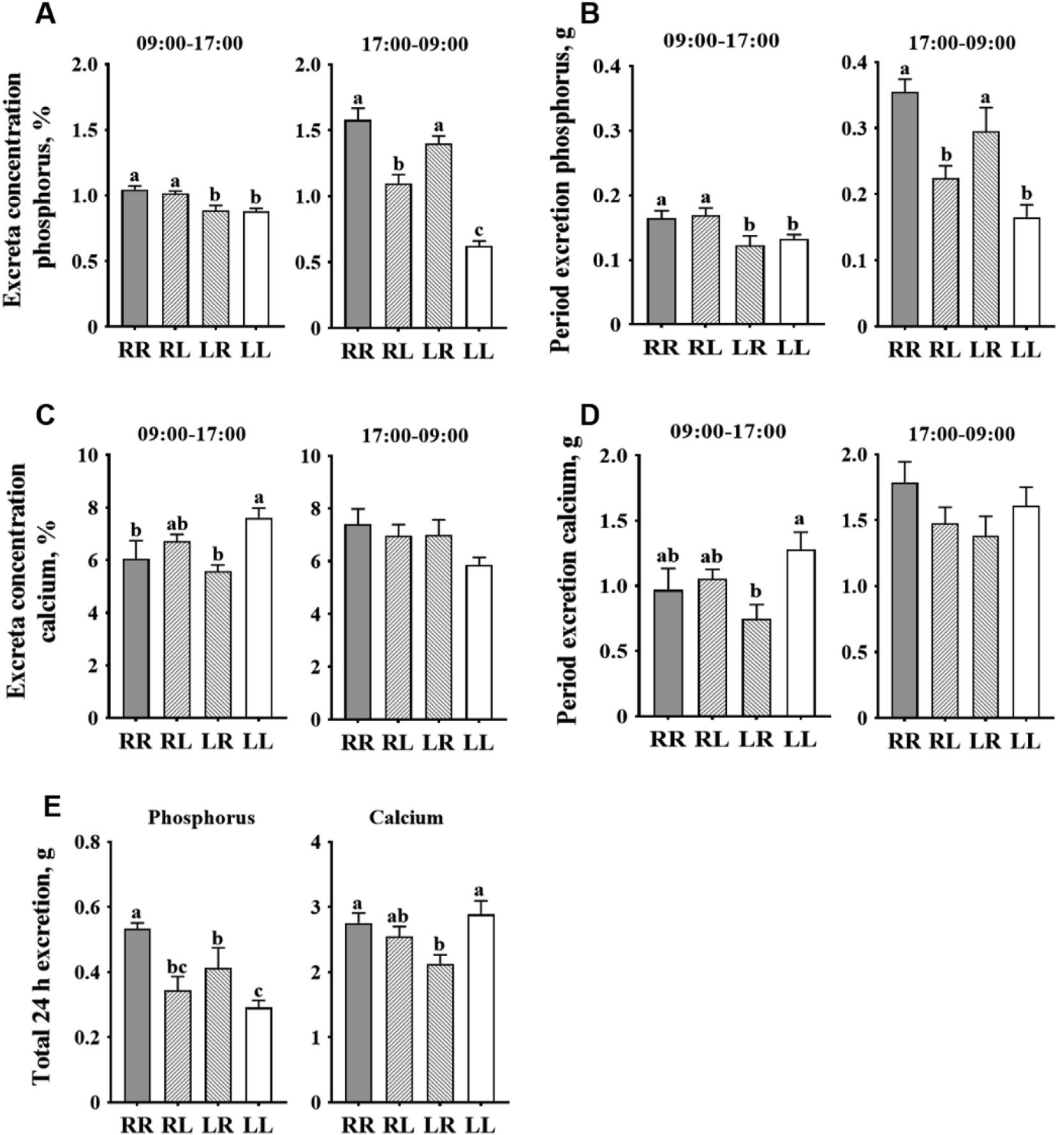
Effects of phosphorus feeding regimens on: (A) excreta phosphorus concentration, (B) period phosphorus excretion, (C) excreta calcium concentration, (D) period calcium excretion, and (E) 24 h total excretion of Hy-Line Brown layers. Four phosphorus feeding regimens: (1) RR, regular phosphorus at both 09:00 and 17:00; (2) RL, regular phosphorus at 09:00 and low phosphorus at 17:00; (3) LR, low phosphorus at 09:00 and regular phosphorus at 17:00; (4) LL, low phosphorus at both 09:00 and 17:00. ^a – c^ Within each sample period, values with no common superscripts differ significantly (*P* < 0.05).

**Fig. 3:**
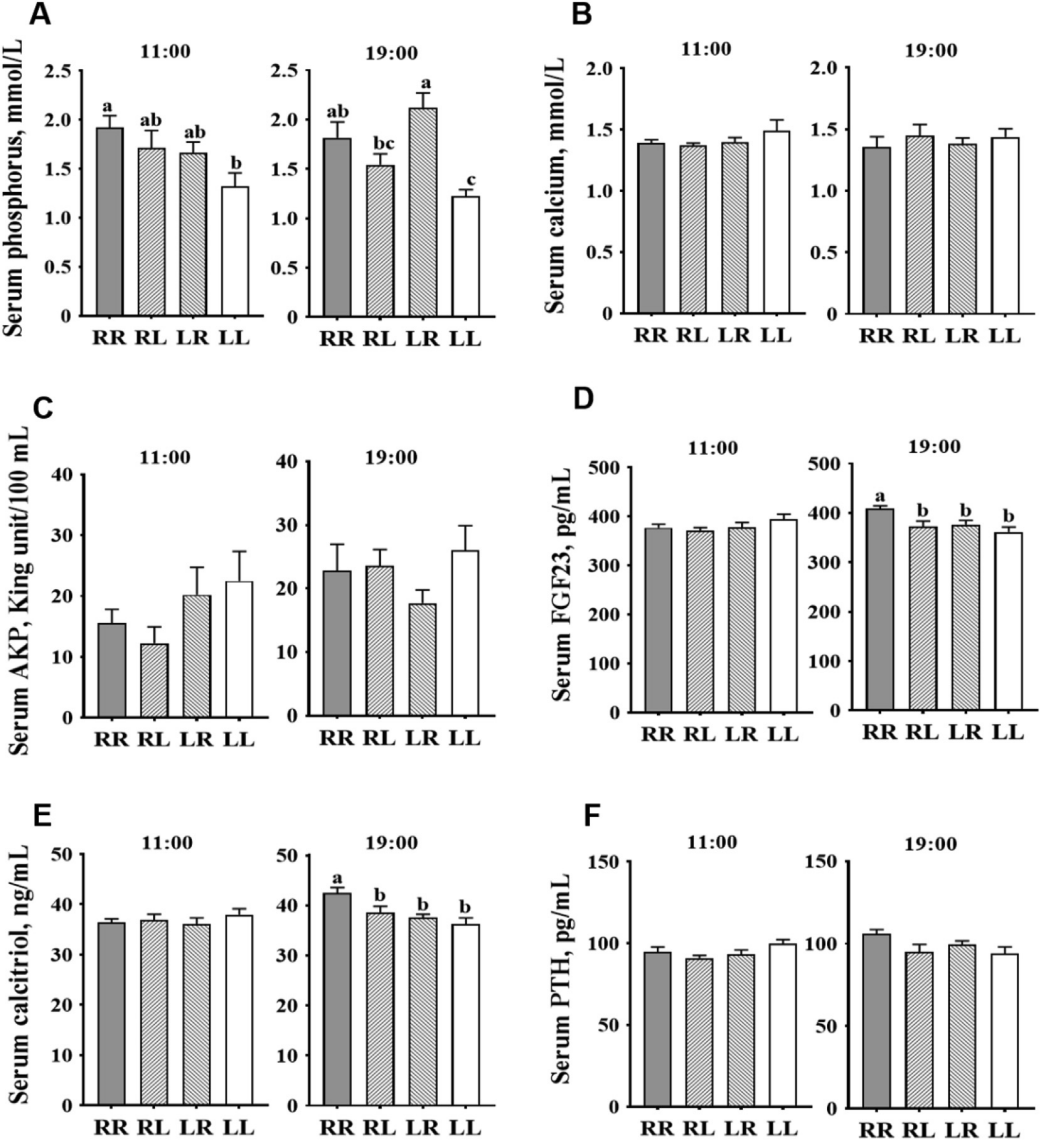
Effects of phosphorus feeding regimens on serum parameters of Hy-Line Brown layers: (A) phosphorus, (B) calcium, (C) AKP, alkaline phosphatase, (D) FGF23, fibroblast growth factor 23, (E) calcitriol, and (F) PTH, parathyroid hormone. Four phosphorus feeding regimens: (1) RR, regular phosphorus at both 09:00 and 17:00; (2) RL, regular phosphorus at 09:00 and low phosphorus at 17:00; (3) LR, low phosphorus at 09:00 and regular phosphorus at 17:00; (4) LL, low phosphorus at both 09:00 and 17:00. ^a – c^ Within each sample time point, values with no common superscripts differ significantly (*P* < 0.05).

During the period of 09:00 to 17:00: (1) layers on the LR and LL phosphorus feeding regimens had decreased phosphorus excretion (Fig. 2A and B; *P* < 0.05, both dry excreta phosphorus concentration and total phosphorus excretion) when compared to those on the regimens of RR and RL; (2) layers on the RR and LR phosphorus feeding regimens had decreased dry excreta calcium concentration (Fig. 2C, *P* < 0.05) when compared to those on the LL regimen; (3) layers on the LR phosphorus feeding regimen had decreased total calcium excretion (Fig. 2D, *P* < 0.05) when compared to those on the LL regimen. During the period of 17:00 to 09:00, layers on the RL and LL phosphorus feeding regimens had decreased phosphorus excretion (Fig. 2A and B; *P* < 0.05, both dry excreta phosphorus concentration and total phosphorus excretion) when compared to those on the regimens of RR and LR. Over the total period of 24 h (09:00 to 09:00 next morning): (1) layers on the RR phosphorus feeding regimen had increased phosphorus excretion when compared to those on the RL, LR, and LL regimens (Fig. 2E); (2) layers on the LR phosphorus feeding regimen had increased phosphorus excretion when compared to those on the LL regimen (Fig. 2E); (3) layers on the LR phosphorus feeding regimen had decreased calcium excretion when compared to those on the RR and LL regimens (Fig. 2E).

### 3.3 Serum and tibiotarsus responses

At 11:00 (2 h after feeding in the morning): (1) layers on the LL phosphorus feeding regimen had decreased serum phosphorus (Fig. 3A, *P* < 0.05) when compared to those on the RR regimen; (2) no differences (*P* > 0.05) were observed on serum calcium (Fig. 3B), AKP (Fig. 3C), FGF23 (Fig. 3D), calcitriol (Fig. 3E) and PTH (Fig. 3F) levels among the 4 different phosphorus feeding regimens. At 19:00 (2 h after feeding in the afternoon): (1) layers on the LR phosphorus feeding regimen had increased serum phosphorus (Fig. 3A, *P* < 0.05) when compared to those on the RL and LL regimens; (2) layers on the RR phosphorus feeding regimen had increased (*P* < 0.05) serum FGF23 (Fig. 3D) and calcitriol (Fig. 3E) levels when compared to those on the regimens of RL, LR and LL. Phosphorus feeding regimens had no effects (Appendix Table 4, *P* > 0.05) on tibiotarsus calcium and phosphorus contents.

### 3.4 Gene and protein expressions of NPT2b, PIT1, PIT2, and CALB1 in the jejunum

**Fig. 4:**
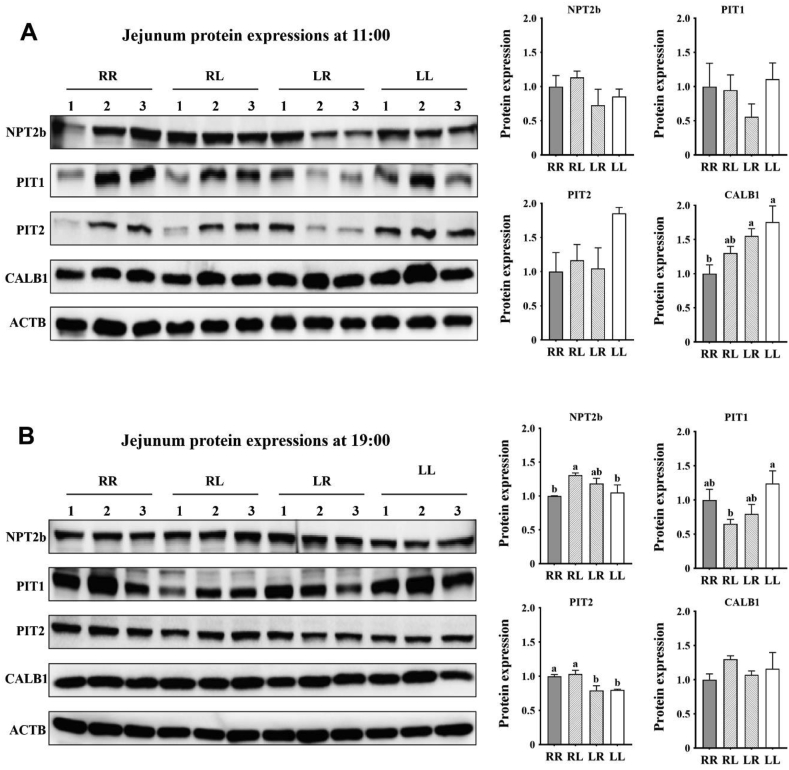
Effects of phosphorus feeding regimens on jejunal protein expressions of Hy-Line Brown layers: (A) protein expressions at 11:00, (B) protein expressions at 19:00. NPT2b = type IIb sodium-phosphate cotransporter; PIT1 = type III sodium-phosphate cotransporter 1; PIT2 = type III sodium-phosphate cotransporter 2; CALB1 = calbindin D28k; ACTB = actin beta. Four phosphorus feeding regimens: (1) RR, regular phosphorus at both 09:00 and 17:00; (2) RL, regular phosphorus at 09:00 and low phosphorus at 17:00; (3) LR, low phosphorus at 09:00 and regular phosphorus at 17:00; (4) LL, low phosphorus at both 09:00 and 17:00. ^a, b^ Within each sample time point, values with no common superscripts differ significantly (*P* < 0.05).

At 11:00 (2 h after feeding in the morning): (1) layers on the RR phosphorus feeding regimen had decreased jejunal CALB1 protein expression (Fig. 4A, *P* < 0.05) when compared to those on the LR and LL regimens; (2) no differences (Appendix Fig. 1A, *P* > 0.05) were observed on jejunal gene expressions. At 19:00 (2 h after feeding in the afternoon): (1) layers on the RL phosphorus feeding regimen had increased jejunal NPT2b protein expression (Fig. 4B**,** *P* < 0.05) when compared to those on the RR and LL regimens, but had decreased jejunal PIT1 protein expression (*P* < 0.05) when compared to those on the LL regimen; (2) layers on the LR and LL phosphorus feeding regimens had decreased jejunal PIT2 protein expression (*P* < 0.05) when compared to those on the RR and RL regimens, and had decreased jejunal *PIT2* gene expression (Appendix Fig. 1B, *P* < 0.05) when compared to those on the RR regimen.

### 3.5 Gene and protein expressions of NPT2a, PIT1, PIT2, and CALB1 in the kidney

**Fig. 5:**
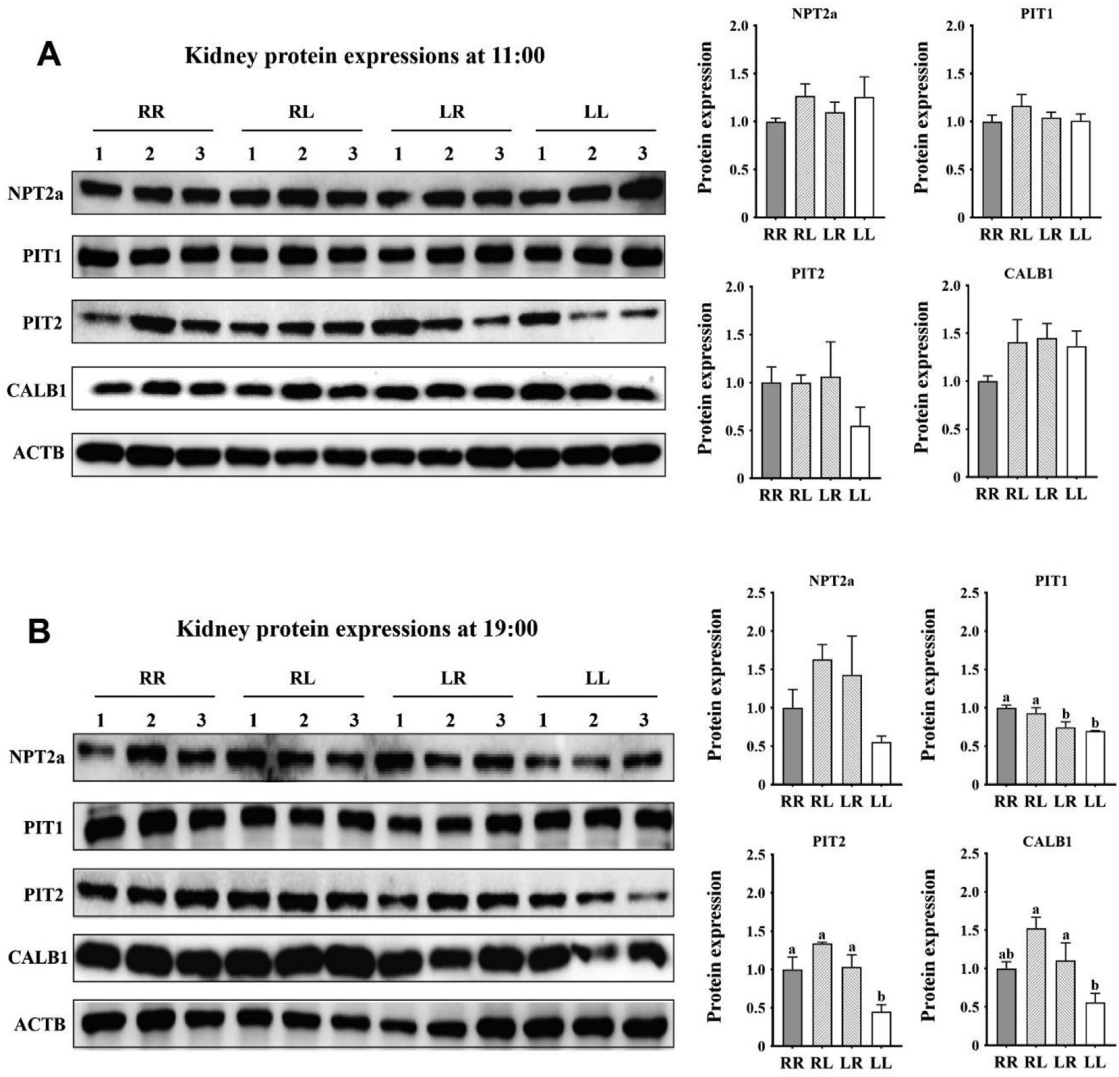
Effects of phosphorus feeding regimens on kidney protein expressions of Hy-Line Brown layers: (A) protein expressions at 11:00, (B) protein expressions at 19:00. NPT2a = type IIa sodium-phosphate cotransporter; PIT1 = type III sodium-phosphate cotransporter 1; PIT2 = type III sodium-phosphate cotransporter 2; CALB1 = calbindin D28k; ACTB = actin beta. Four phosphorus feeding regimens: (1) RR, regular phosphorus at both 09:00 and 17:00; (2) RL, regular phosphorus at 09:00 and low phosphorus at 17:00; (3) LR, low phosphorus at 09:00 and regular phosphorus at 17:00; (4) LL, low phosphorus at both 09:00 and 17:00. ^a, b^ Within each sample time point, values with no common superscripts differ significantly (*P* < 0.05).

At 11:00 (2 h after feeding in the morning), no differences (Appendix Fig. 2A, *P* > 0.05) were observed on kidney gene expressions and protein expressions. At 19:00 (2 h after feeding in the afternoon): (1) layers on the RR and RL phosphorus feeding regimens had increased kidney PIT1 protein expression (Fig. 5B, *P* < 0.05) when compared to those on the LR and LL regimens; (2) layers on the LL phosphorus feeding regimen had decreased kidney PIT2 protein expression (*P* < 0.05) when compared to those on the RR, RL and LR regimens, and had decreased kidney CALB1 protein expression (*P* < 0.05) when compared to those on the RL and LR regimens; (3) layers on the RL phosphorus feeding regimens had increased kidney *CALB1* gene expression (Appendix Fig. 2B, *P* < 0.05) when compared to those on the LR and LL regimens.

## 4 Discussion

The current results support the hypothesis that a simple daily dynamic feeding regimen may help to reduce the consumption and excretion of phosphorus in laying hens without negative effects on egg production performance (MolnÁR et al., 2018). Namely, laying hens fed with the LR phosphorus regimen (low phosphorus diet at 09:00 and regular phosphorus diet at 17:00) had similar laying performance and eggshell quality when compared to those fed with the RR phosphorus regimen (regular phosphorus diet at both 09:00 and 17:00). If so, the laying hen industry could save a significant amount of inorganic phosphate consumption with application of the LR phosphorus regimen (laying hens consume about 380,000 tons of inorganic phosphate each year in China). The LL phosphorus regimen (low phosphorus diet at both 09:00 and 17:00) represents a direct deprivation of dietary inorganic phosphate without considering the daily egg-laying cycle. Even though the LL phosphorus regimen had no effect on the mineralization status of the tibiotarsus, the decreased eggshell quality in the LL phosphorus regimen group indicates that the supplementation of inorganic phosphate is required to some extent, at least under the current experimental conditions. Indeed, the possibility of feeding laying hens with diets containing no inorganic phosphate has long been discussed. There is data published that showed laying hens can produce normally from 20 to 70 wk of age when fed with a corn-soybean meal-based diet containing 0.15% available phosphorus (Boling et al., 2000). The inconsistent observations may have resulted from alterations in factors like the age of laying hens and calcium and vitamin D levels, indicating the potential risk of simply depriving inorganic phosphate from laying hen diets (Teng et al., 2020).

Of particular interest, the RL phosphorus regimen (regular phosphorus diet at 09:00 and low phosphorus diet at 17:00), which was hypothesized to precisely meet the dynamic phosphorus requirements during the daily egg-laying cycle, caused significant reduction of egg laying rate starting in wk 3 and lasting to the end of the feeding trial. During the eggshell formation period, a high concentration of serum phosphorus is physiologically required for maintaining normal egg laying performance. Counteracting the circadian rhythm of phosphorus metabolism by supplying a low phosphorus diet may lead to unexpected laying disorders. On the contrary, working synchronously with serum phosphorus rhythm (Kerschnitzki et al., 2014), using the LR phosphorus feeding regimen, may be a potential direction to consider to effectively manage phosphorus turnover in laying hens. These observations warrant long-term studies with increased animal numbers to understand the mechanisms of serum phosphate rhythms and develop field-applicable daily dynamic feeding techniques in laying hens. In the current study, the phosphorus feeding regimens were designed based on a commercial feeding practice (all the laying hens were fed at the same time, twice daily, at 09:00 and 17:00) without considering the egg-laying cycle of each laying hen. The consumption of phosphorus will be better managed if the diets could be supplemented according to the serum phosphorus rhythm of each individual laying hen in future feeding systems.

In the current study, the dietary non-phytate phosphorus level (0.32%) for the RR phosphorus feeding regimen was designed according to the specification of the Chinese Feeding Standard of Chicken (NY/T 33-2004), which represents the most common practice in the current laying hen industry. The LR phosphorus feeding regimen decreased the amount of phosphorus excretion by 26% during the period of 09:00 to 17:00, by 17% during the period of 17:00 to 09:00, and by 22% over the total period of 24 h, highlighting the potential value of dynamic phosphorus feeding in both phosphate rock resource saving and pollution reduction (Fink et al., 2018; Mallin, 2000; Vaccari et al., 2019). Unfortunately, we did not measure the feed intake of those hens over the 24 h excreta collection period. Although no difference was observed on feed intake among treatments throughout the feeding trial, the laying hens selected for excreta collection may differ in feed intake. Studies with prolonged excreta collection periods and precise feed consumption recordings will be essential for further confirming how much phosphorus can be saved and to what extent excretion can be reduced by using daily dynamic phosphorus feeding regimens. Also, because feed intake and excretion are not equally distributed throughout the day, more time points during the day may need to be monitored in future studies.

FGF23 plays a central role in setting up the physiological ceiling for phosphorus concentration in the circulation (Crenshaw et al., 2011; Edmonston and Wolf, 2020). In the current study, limiting dietary phosphorus influx, by feeding with either RL or LR or LL phosphorus regimens, significantly decreased serum FGF23 levels and subsequently mitigated body phosphorus loss during the eggshell formation period. Obviously, the secretion of FGF23 is more likely to be induced to higher levels by medullary bone resorption (Whitehead, 2004) when the laying hens are consistently supplied with high phosphorus diets. An important observation was that, laying hens on the LR phosphorus feeding regimen achieved higher levels of serum phosphorus (indicating a positive enhancement of phosphorus rhythms), however, these hens exhibited lower levels of serum FGF23 (indicating a decrease of body phosphorus loss), during the eggshell formation period, when compared to the RR regimen. These results, along with the findings on egg production performance, inferred that future phosphorus-reduction strategies (e.g., dynamic feeding regimens) need to be developed to effectively maintain physiological rhythms of serum phosphorus in egg-laying hens (MolnÁR et al., 2018). In other words, fully characterizing the daily phosphorus rhythms of laying hens, and adjusting phosphorus feeding regimens, accordingly, may help maximize the utilization of dietary phosphorus. In the current study, the calcium-phosphorus metabolism status of laying hens was evaluated at 2 time points (11:00, 2 h after feeding in the morning; and 19:00, 2 h after feeding in the afternoon). These sampling time points, which were selected during the period when serum phosphorus acutely increased with feed intake (Lepkovsky et al., 1960; Shirley et al., 1954), demonstrated that daily feeding regimens can effectively interfere with the serum phosphorus rhythms in laying hens. In future studies, evaluating the calcium-phosphorus metabolism status at different times during the post-prandial response, may help to further understand the mechanisms and develop more precise feeding regimens for reducing phosphorus consumptions in the egg production industry. In addition to feed intake, the time of egg-laying will also need to be carefully considered when evaluating the calcium-phosphorus metabolism status of laying hens. In the current study, the sampled laying hens were randomly selected from those that laid eggs before 08:30 in the morning. This may insert variability into measures as not all laying hens laid at the same time. In order to accurately evaluate the post-prandial and post-ovipositional body calcium-phosphorus rhythms, the experimental diets will need to be provided according to the egg-laying time of each individual laying hen in future studies. There was no difference in serum total calcium concentrations among treatments. Since total calcium cannot truly reflect the degree of ionization of calcium, analyzing serum ionized calcium, which has been tightly linked to the bone remodelling process, may help to further illustrate the effects of dynamic feeding regimens.

Body phosphorus homeostasis at the molecular level is accomplished by the coordination of intestinal and renal sodium-phosphate cotransporters (Berndt and Kumar, 2009). In laying hens, the eggshell formation period is a sensitive stage for body phosphorus loss (Prashad and Edwards, 1973). Interestingly, laying hens on the RL phosphorus feeding regimen had increased levels of jejunal NPT2b protein expression during the eggshell formation period. However, when the daily rhythm of serum phosphorus was reversed by feeding phosphorus against serum changes (i.e., feeding low phosphorus diet when serum phosphorus is high and feeding high phosphorus diet when serum phosphorus is low), the laying hens correspondingly adjusted intestinal phosphate transporters to enhance the serum phosphorus rhythm (Chande and Bergwitz, 2018; Yves et al., 2009), which might be required for a normal egg laying performance. Laying hens on the LR phosphorus feeding regimen exhibited low phosphorus influx efficiency, shown as reduced productions of jejunal PiT2 and kidney PiT1 proteins, indicating the dietary non-phytate phosphorus level (0.32%) at 17:00 could be decreased for further reduction of phosphorus consumption and excretion. The protein expression of cellular calcium transporter CALB1 in intestine and renal samples well explained the increased calcium excretion and poor eggshell quality in LL regimen fed laying hens (Diaz de Barboza et al., 2015).

## 5 Conclusions

Two diets with different phosphorus levels were alternately presented to laying hens during different stages of the daily egg-laying cycle. When dietary phosphorus was supplied following the daily serum phosphorus rhythms (i.e., LR regimen in the current study), the laying performance and egg quality were well supported with significant decreases in inorganic phosphate composition and phosphorus excretion. However, when dietary phosphorus was supplied against the daily serum phosphorus rhythms (i.e., RL regimen in the current study), unexpected laying disorders occurred (egg laying rate decreased by 11%). The current results underscore the importance of maintaining serum phosphorus rhythms during the daily eggshell calcification cycle when developing phosphorus-reduction strategies in laying hens. These observations warrant long-term studies with increased animal numbers to develop dynamic feeding regimens for reducing phosphorus consumption and excretion in laying hens.

## Author contributions

**Xujie Liao**: Investigation, Data curation, Formal analysis, Writing-Original draft preparation; **Jiakun Yan**: Investigation, Data curation, Formal analysis; **Jionghao Chen**: Investigation, Data curation; **Zhenyu Huang**: Investigation, Data curation; **Tianshuai Xiao**: Investigation, Data curation; **Changqing Li**: Investigation, Data curation; **Chong Pan**: Investigation, Data curation; **Xin Yang**: Methodology, Formal analysis; **Yanli Liu**: Methodology, Formal analysis; **Thomas D. Crenshaw**: Methodology, Writing-Reviewing and Editing, Supervision; **Xiaojun Yang**: Conceptualisation, Writing-Reviewing and Editing, Supervision; **Zhouzheng Ren**: Conceptualisation, Formal analysis, Writing-Original draft preparation, Writing-Reviewing and Editing, Supervision.

## Declaration of competing interest

The authors declare that they have no competing interests.

## Appendix A Supplementary data

22-00250_Supplementary data_for production.docx 22-00250_Supplementary data_for production.docx
