# Supplementary material for: A simple daily dynamic feeding regimen for reducing phosphorus consumption and excretion in laying hens
Source: Anim Nutr. 2022 Jul 22;11:132–41. doi: 10.1016/j.aninu.2022.07.003 (PMC9527630; doi:10.1016/j.aninu.2022.07.003)
Supplement: 22-00250_Supplementary data_for production.docx [file mmc1.docx]

**Supplementary data**

**Appendix Table 1**

Sources and concentrations of antibodies used in the Western blotting analysis

| Antibody | Host | Dilution | Source and catalog number |
| --- | --- | --- | --- |
| Primary antibody^1^ | | | |
| ACTB | Mouse | 1:1,000 | CWBIO Co., Ltd. (Beijing, China); CW0096 |
| NPT2a | Rabbit | 1:1,000 | ABclonal Technology (Wuhan, China); A6742 |
| NPT2b | Rabbit | 1:1,000 | ABclonal Technology (Wuhan, China); A9460 |
| PIT1 | Rabbit | 1:1,000 | ABclonal Technology (Wuhan, China); A4117 |
| PIT2 | Rabbit | 1:1,000 | ABclonal Technology (Wuhan, China); A6739 |
| CALB1 | Rabbit | 1:1,000 | ABclonal Technology (Wuhan, China); A15035 |
|  |  |  |  |
| Secondary antibody |  |  |  |
| Goat anti-rabbit |  | 1:1,000 | Diyi Biotechnology Co., Ltd. (Hubei, China); DY60202 |
| Goat anti-mouse |  | 1:1,000 | Bioss Biotechnology Co., Ltd. (Beijing, China); bs-0296G-HRP |

^1^ACTB, actin beta; NPT2a, type IIa sodium-phosphate cotransporter; NPT2b, type IIb sodium-phosphate cotransporter; PIT1, type III sodium-phosphate cotransporter 1; PIT2, type III sodium-phosphate cotransporter 2; CALB1, calbindin D28k*.*

**Appendix Table 2**

Sequences of primers used for the quantitative real-time PCR analysis

| Gene^1^ | NCBI accession no. | Primer (5'-3')^2^ |
| --- | --- | --- |
| *ACTB* | NM_205518.2 | F: AATCAAGATCATTGCCCCACCT |
|  |  | R: TGGGTGTTGGTAACAGTCCG |
| *NPT2a* | XM_015293846.2 | F: CCAAACTGCACGGCTTCT |
|  |  | R: TGGGAGGTCAGTGTTGATGA |
| *NPT2b* | NM_204474.2 | F: GCCTGGTAAAGGTTTGGTGC |
|  |  | R: TGCCGGCATTTAGTGATATATTCTG |
| *PIT1* | XM_040689632.1 | F: GCTCGTGGCTTCGTTCTTG |
|  |  | R: GGACCATTTGACGCCTTTCT |
| *PIT2* | NM_001305398.2 | F: GCAGCAGATACATCAACTC |
|  |  | R: ATTTCCACTCCACCCTC |
| *CALB1* | NM_205513.2 | F: CAGGGTGTCAAAATGTGTGC |
|  |  | R: GCCAGTTCTGCTCGGTAAAG |

*^1^ACTB*, actin beta; *NPT2a*, type IIa sodium-phosphate cotransporter; *NPT2b*, type IIb sodium-phosphate cotransporter; *PIT1*, type III sodium-phosphate cotransporter 1; *PIT2*, type III sodium-phosphate cotransporter 2; *CALB1*, calbindin D28k.

^2^F, forward; R, reverse.

**Appendix Table 3**

Effect of phosphorus feeding regimens on egg quality of Hy-Line Brown layers

| Item | Time of dietary treatment, wk | Daily phosphorus regimen^1^ | | | | SEM | *P*-value |
| --- | --- | --- | --- | --- | --- | --- | --- |
|  |  | RR | RL | LR | LL |  |  |
| Shell index, % of whole egg | 0 | 11.2 | 11.3 | 11.6 | 11.1 | 0.1 | 0.209 |
|  | 4 | 11.4 | 11.8 | 11.6 | 11.4 | 0.1 | 0.636 |
|  | 8 | 12.1 | 12.0 | 12.4 | 11.4 | 0.2 | 0.206 |
|  | 12 | 11.4 | 11.1 | 11.3 | 10.7 | 0.1 | 0.215 |
|  |  |  |  |  |  |  |  |
| Haugh units | 0 | 86.0 | 88.0 | 81.6 | 83.3 | 1.9 | 0.668 |
|  | 4 | 87.4 | 85.4 | 88.3 | 84.3 | 1.6 | 0.804 |
|  | 8 | 88.7 | 82.3 | 85.3 | 82.3 | 2.0 | 0.631 |
|  | 12 | 75.7 | 81.5 | 80.8 | 84.1 | 1.7 | 0.207 |
|  |  |  |  |  |  |  |  |
| Yolk pigmentation | 0 | 6.6 | 6.6 | 6.4 | 6.3 | 0.1 | 0.474 |
|  | 4 | 6.9 | 6.8 | 6.6 | 6.5 | 0.1 | 0.607 |
|  | 8 | 6.6 | 6.6 | 6.7 | 6.5 | 0.1 | 0.817 |
|  | 12 | 6.4 | 6.4 | 6.7 | 6.8 | 0.1 | 0.108 |

^1^Four phosphorus feeding regimens: (1) RR, regular phosphorus at both 09:00 and 17:00; (2) RL, regular phosphorus at 09:00 and low phosphorus at 17:00; (3) LR, low phosphorus at 09:00 and regular phosphorus at 17:00; (4) LL, low phosphorus at both 09:00 and 17:00.

**Appendix Table 4**

Effects of phosphorus feeding regimens on tibiotarsus calcium and phosphorus contents of Hy-Line Brown layers

| Item | Daily phosphorus regimen^1^ | | | | SEM | *P*-value |
| --- | --- | --- | --- | --- | --- | --- |
|  | RR | RL | LR | LL |  |  |
| Calcium content, % of ash | 41.18 | 44.14 | 43.97 | 41.87 | 0.85 | 0.545 |
| Total calcium, g | 1.58 | 1.63 | 1.56 | 1.55 | 0.03 | 0.890 |
| Phosphorus content, % of ash | 19.57 | 21.61 | 20.82 | 19.38 | 0.44 | 0.228 |
| Total phosphorus, g | 0.75 | 0.79 | 0.74 | 0.72 | 0.02 | 0.341 |
| Calcium-to-phosphorus ratio, g:g | 2.11 | 2.04 | 2.12 | 2.16 | 0.02 | 0.377 |

^1^Four phosphorus feeding regimens: (1) RR, regular phosphorus at both 09:00 and 17:00; (2) RL, regular phosphorus at 09:00 and low phosphorus at 17:00; (3) LR, low phosphorus at 09:00 and regular phosphorus at 17:00; (4) LL, low phosphorus at both 09:00 and 17:00.

**Appendix Fig. 1**


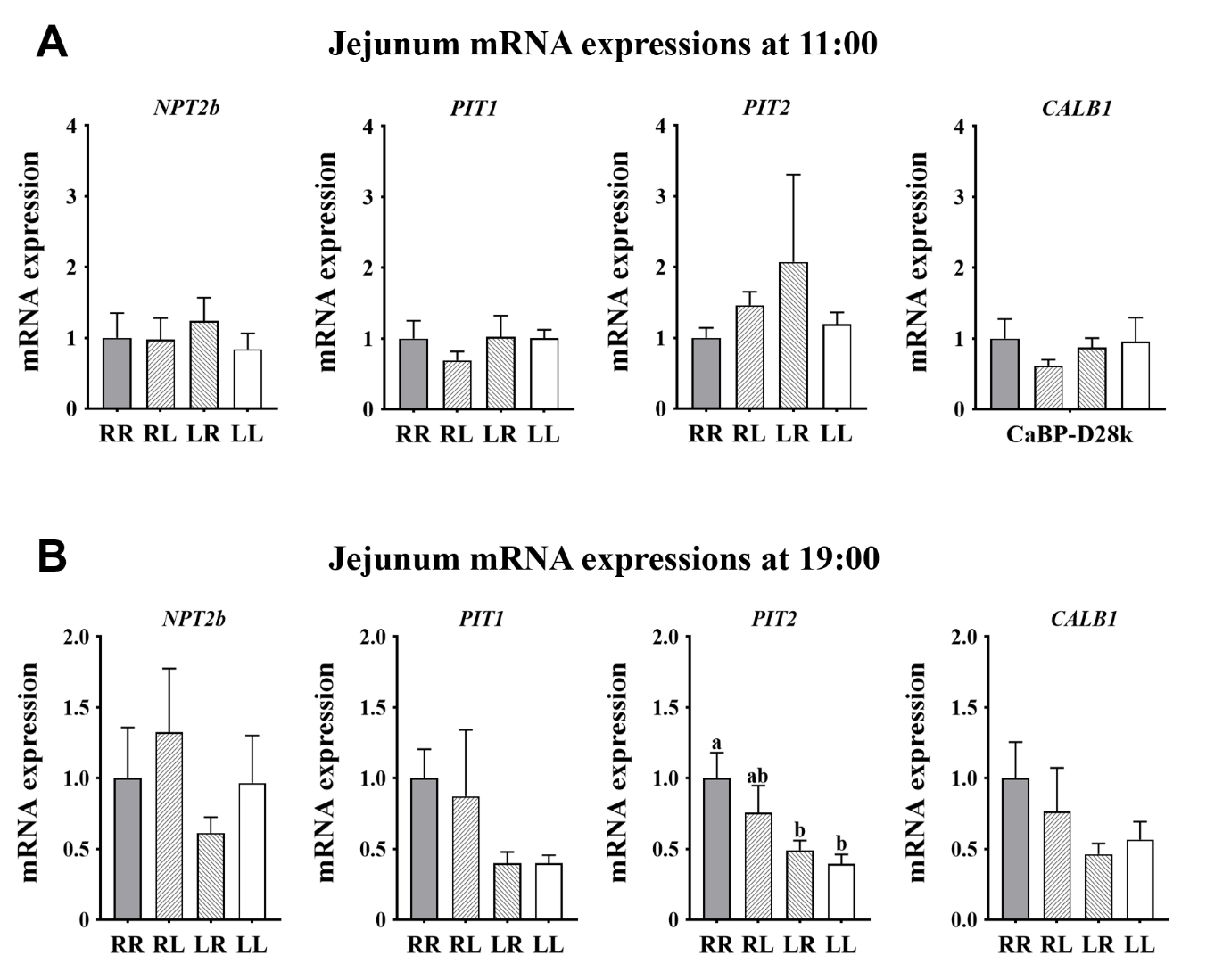


**Appendix Fig. 1.** Effects of phosphorus feeding regimens on jejunal mRNA expressions of Hy-Line Brown layers: (A) mRNA expressions at 11:00, (B) mRNA expressions at 19:00.*NPT2b* = type IIb sodium-phosphate cotransporter; *PIT1* = type III sodium-phosphate cotransporter 1; *PIT2* = type III sodium-phosphate cotransporter 2; *CALB1* = calbindin D28k; *ACTB* = actin beta. Four phosphorus feeding regimens: (1) RR, regular phosphorus at both 09:00 and 17:00; (2) RL, regular phosphorus at 09:00 and low phosphorus at 17:00; (3) LR, low phosphorus at 09:00 and regular phosphorus at 17:00; (4) LL, low phosphorus at both 09:00 and 17:00. ^a, b^ Within each sample time point, values with no common superscripts differ significantly (*P* < 0.05).

**Appendix Fig. 2**


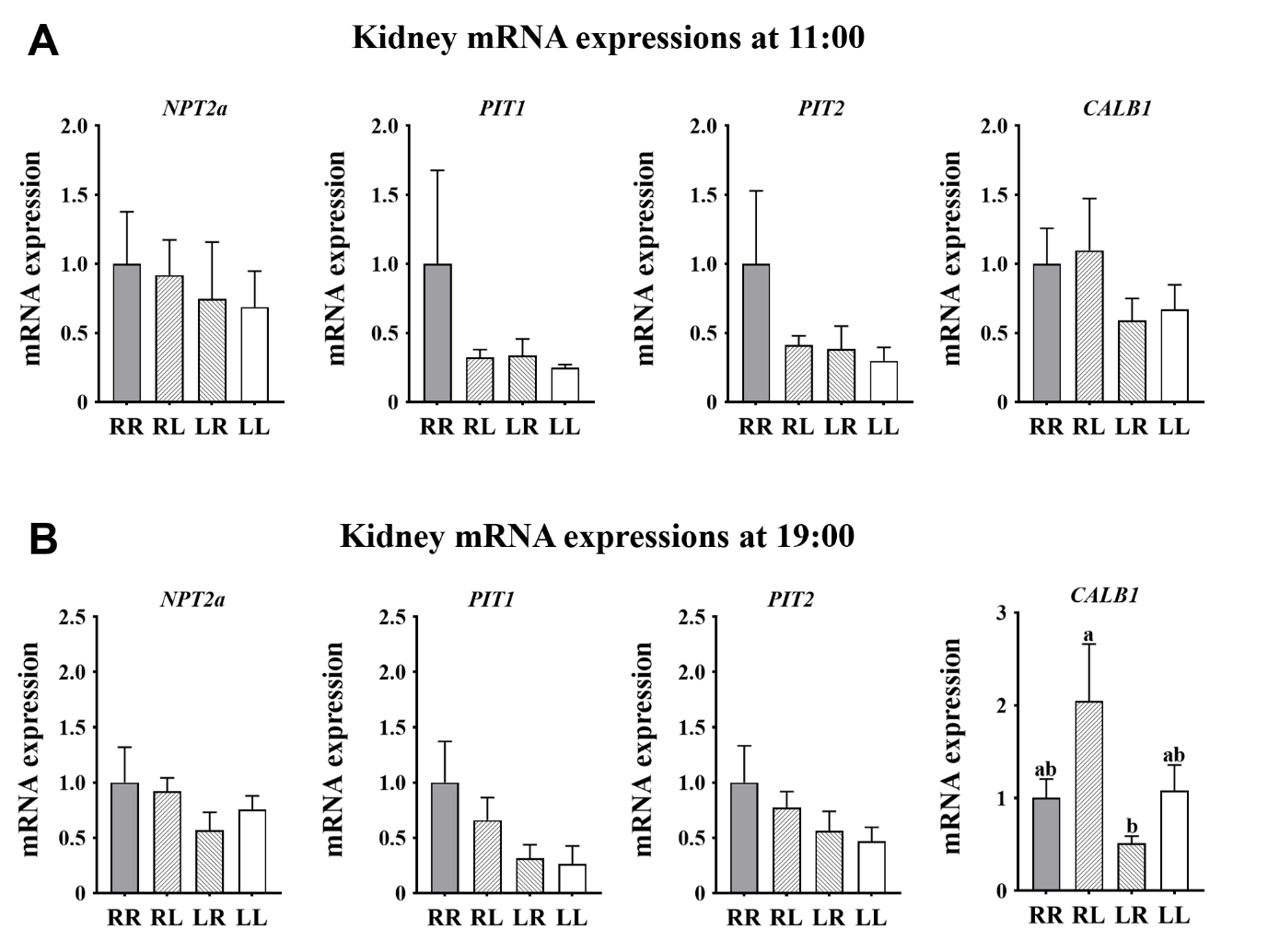


**Appendix Fig. 2.** Effects of phosphorus feeding regimens on kidney mRNA expressions of Hy-Line Brown layers: (A) mRNA expressions at 11:00; (B) mRNA expressions at 19:00. *NPT2a* = type IIa sodium-phosphate cotransporter; *PIT1* = type III sodium-phosphate cotransporter 1; *PIT2* = type III sodium-phosphate cotransporter 2; *CALB1* = calbindin D28k; *ACTB* = actin beta. Four phosphorus feeding regimens: (1) RR, regular phosphorus at both 09:00 and 17:00; (2) RL, regular phosphorus at 09:00 and low phosphorus at 17:00; (3) LR, low phosphorus at 09:00 and regular phosphorus at 17:00; (4) LL, low phosphorus at both 09:00 and 17:00. ^a, b^ Within each sample time point, values with no common superscripts differ significantly (*P* < 0.05).
